# New Insights in Thyroid Cancer and p53 Family Proteins

**DOI:** 10.3390/ijms18061325

**Published:** 2017-06-21

**Authors:** Livia Manzella, Stefania Stella, Maria Stella Pennisi, Elena Tirrò, Michele Massimino, Chiara Romano, Adriana Puma, Martina Tavarelli, Paolo Vigneri

**Affiliations:** 1 Department of Clinical and Experimental Medicine, University of Catania, 95124 Catania, Italy; stefania.stel@gmail.com (S.S.); perny76@gmail.com (M.S.P.); ele_tir@yahoo.it (E.T.); michedot@yahoo.it (M.M.); chiararomano83@libero.it (C.R.); adry.p88@hotmail.it (A.P.); pvigneri@libero.it (P.V.); 2 Endocrinology, Department of Clinical and Experimental Medicine, Garibaldi Nesima Medical Center, University of Catania, 95122 Catania, Italy; martinatava@hotmail.it

**Keywords:** thyroid cancer, p53, p63, p73, genetic alterations, p53 inhibition mechanisms, target therapies

## Abstract

Thyroid cancers are common endocrine malignancies that comprise tumors with different clinical and histological features. Indeed, papillary and follicular thyroid cancers are slow-growing, well-differentiated tumors, whereas anaplastic thyroid cancers are undifferentiated neoplasias that behave much more aggressively. Well-differentiated thyroid carcinomas are efficiently cured by surgery and radioiodine, unlike undifferentiated tumors that fail to uptake radioactive iodine and are usually resistant to chemotherapy. Therefore, novel and more effective therapies for these aggressive neoplasias are urgently needed. Whereas most genetic events underlying the pathogenesis of well-differentiated thyroid cancers have been identified, the molecular mechanisms that generate undifferentiated thyroid carcinomas are still unclear. To date, one of the best-characterized genetic alterations leading to the development of poorly differentiated thyroid tumors is the loss of the p53 tumor suppressor gene. In addition, the existence of a complex network among p53 family members (p63 and p73) and their interactions with other factors that promote thyroid cancer progression has been well documented. In this review, we provide an update on the current knowledge of the role of p53 family proteins in thyroid cancer and their possible use as a therapeutic target for the treatment of the most aggressive variants of this disease.

## 1. Introduction

Thyroid cancer is the most common endocrine neoplasm, accounting for about 1.7% of total cancer diagnoses each year in the USA [1]. Worldwide, disease incidence has increased three-fold over the past 30 years because of both a higher prevalence in thyroid gland screening and environmental and life-style changes [2]. The risk of developing a thyroid tumor depends on genetic factors, age, gender, histological type, radiation exposure, and geographical region [3]. Neoplastic transformation of the follicular epithelium generates well-differentiated thyroid cancers (WDTC), including papillary (PTC) and follicular (FTC) thyroid carcinomas, and undifferentiated tumors represented by poorly differentiated (PDTC) and anaplastic thyroid carcinomas (ATC). PTCs and FTCs account for 85% and 10% of all thyroid carcinomas, respectively, with the remaining tumors unevenly distributed between PDTCs and ATCs [4].

The treatment for WDTC is based on surgery, radioactive iodine, and thyroid hormone therapy [5]. On the other hand, PDTCs or ATCs are refractory to hormone therapy and lack expression of the Sodium/Iodide Symporter (NIS) required for radioactive iodine uptake. Moreover, although metastases are observed in 10 to 20% of patients with ATC, most of them succumb to locally invasive, inoperable disease [6]. Furthermore, ATC is usually refractory to conventional chemotherapy [6] with more than 50% of affected individuals dying within one year from diagnosis [7].

**Figure 1:**
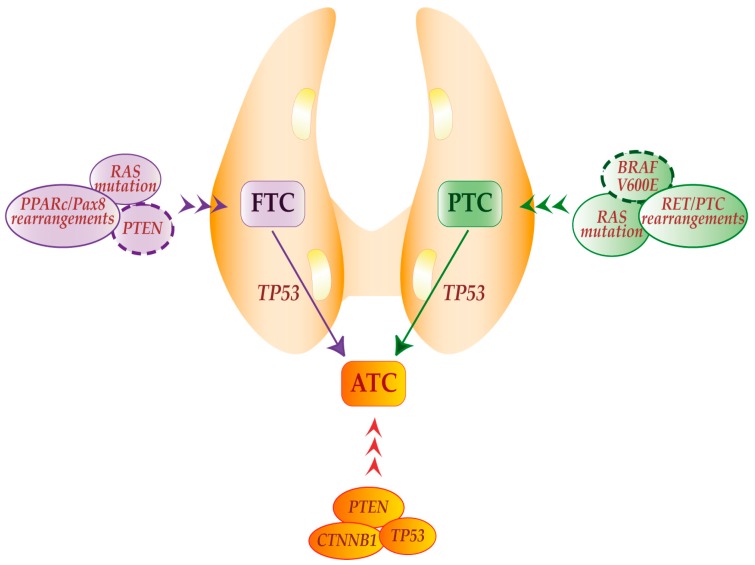
Schematic representation of thyroid cancer hystotypes and their causative genetic events. Papillary (PTC), Follicular (FTC) and Anaplastic Thyroid carcinomas (ATC) originate from thyroid follicular cells. PTCs display BRAF (V600E substitution) and/or Rat Sarcoma (*RAS*) mutations as well as Rearrangement During Transfection (*RET*)*/PTC* rearrangements. FTCs present *PPARc/Pax8* rearrangements, *RAS*, mutations and *PTEN* inactivating mutations or deletions. ATCs are characterized by *PTEN* and *CTNNB1* mutations and p53 inactivation. Furthermore, ATCs may arise from FTCs and PTCs as a result of p53 loss of function, dysregulation of the PTEN/PI3K/AKT pathway or additional genetic alterations. Dashed lines indicate genes and mechanisms involved in progression to ATC.

Multiple studies have improved our understanding of the mechanisms underlying thyroid carcinogenesis. Presently, we know that several forms of thyroid cancer are characterized by mutually exclusive somatic mutations and/or gene rearrangements, which trigger increased cell proliferation and survival [8,9,10]. PTCs are often characterized by activating mutations of the BRAF (V600E substitution) that have been reported in up to 70% of cases. Rat Sarcoma (*RAS*) point mutations in codons 12, 13, and 61 and rearrangements of the Rearrangement During Transfection (*RET*) gene (generating *RET/PTC* chimeric oncogenes) represent other frequent genetic alterations (Figure 1). These events share a common downstream signaling pathway as they lead to the improper activation of the mitogen-activated protein kinase (MAPK) [11].

FTCs display a different genetic profile (Figure 1) characterized by the presence of peroxisome proliferator-activated receptor c/paired box 8 (*PPARc/Pax8*) genetic rearrangements (30%), *RAS* activating point mutations (20–40%) and *PTEN* inactivation by point mutations, deletions, or promoter methylation (<10%) [12].

On the other hand, ATCs can be initiated by the coexistence of activating mutations in *CTNNB1* (β-catenin encoding gene) and inactivation of both *p53* and *PTEN* [13]. In addition, ATCs may arise from WDTC when *PTEN* inactivation (in the case of FTC) or *BRAF* activation (in the case of PTC) are associated with *p53* loss of function (Figure 1). Finally, ATC may display mutations in the catalytic subunit alpha of the phosphatidyl-inositol-3-kinase (PIK3CA) that, in addition to PTEN inactivation, cause constitutive activation of the AKT pathway resulting in a more aggressive phenotype [14].

These alterations represent prognostic markers associated with tumor progression and potential targets that could become therapeutically actionable in the near future. In this review we intend to clarify the role of the p53 protein family in thyroid tumorigenesis. Furthermore we provide an update on the new therapeutic approaches involving p53 for the treatment of the most aggressive variants of the disease.

## 2. The p53 Protein Family

**Figure 2:**
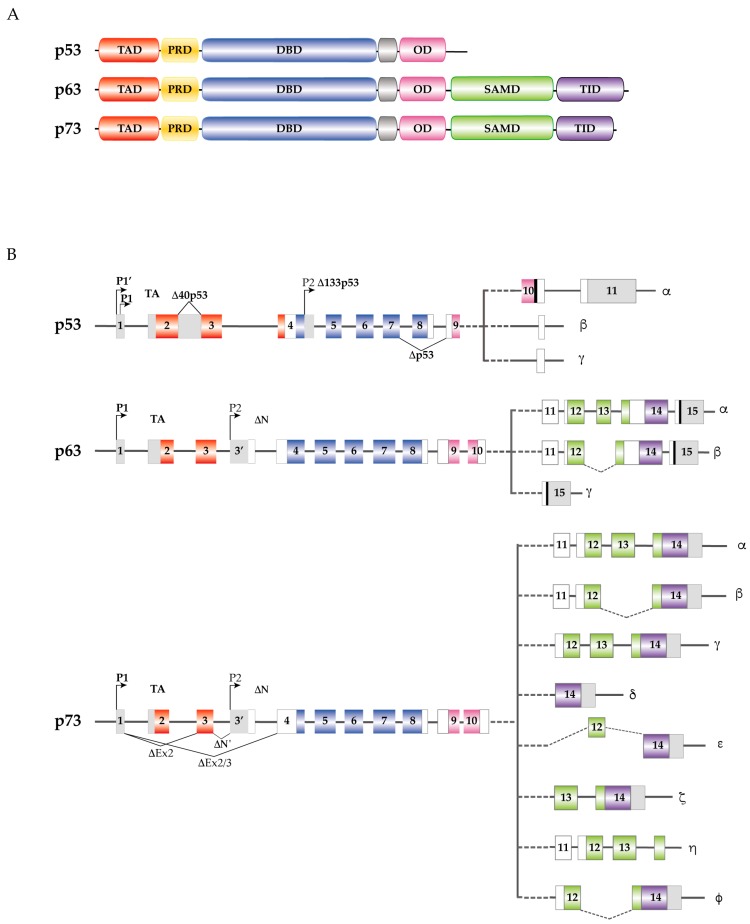
p53 family proteins and isoforms. (**A**) Functional domains of the p53 family members. Red, Transactivation Domain (TAD); yellow, Proline-Rich sequence Domain (PRD); blue, DNA Binding Domain (DBD); pink, Oligomerization Domain (OD); green, Sterile α Motif Domain (SAMD); purple, Transactivation Inhibitory Domain (TID); (**B**) *p53*, *p63* and *p73* intron/exon structure. Introns are depicted in gray, while exon coloring reflects the corresponding functional domains. All three genes express multiple splice variants and contain different internal promoters. p53 includes TAp53, Δ40p53 (generated by an alternative splicing of intron 2), Δp53 (produced by alternative splicing of exons 7/9), and Δ133p53 (generated using an internal promoter in intron 4). The alternative splicing of intron 9 gives rise to α, β, and γ isoforms. In *p63*, the proximal P1 promoter yields the TA isoforms, while the distal P2 promoter in intron 3′, gives rise to ΔNp63 truncated variants. In addition, the COOH-terminal splicing leads to p63 α, β and γ isoforms for both the TA and ΔN variants. As for p73 the P1 promoter generates the TA isoforms, while the P2 distal promoter in intron 3′, gives rise to *ΔNp73* truncated variants. Moreover, p73 can use an additional NH2-terminal splicing site, within exon 2, that produces ΔN like proteins Ex2p73, Ex2/3p73 and ΔN’p73. The COOH-terminal splicing leads to p73 α, β, γ, δ, ε, ζ, η, and ϕ isoforms for both TA and ΔN variants.

*p53*, *p63* and *p73* are tumor suppressor genes encoding for proteins with high homology. The common protein domains consist of an amino-terminal transactivation domain (TAD), a central DNA binding domain (DBD), a carboxy-terminal oligomerization domain (OD), and a proline-rich sequence recognition domain (PRD) [15] (Figure 2A).

The TAD is the binding site for several transcription regulators [16]. The DBD mediates binding to target DNA sequences [17] while the OD induces oligomers formation that influence DNA-binding and transcriptional activity [18]. In addition to the aforementioned domains, p63 and p73 present a sterile α motif domain (SAMD) that modulates protein-protein interactions [19,20] and a transactivation inhibitory domain (TID) that regulates their transcription activity [21] (Figure 2A).

*p53*, *p63* and *p73* express different internal promoters located at the amino-terminus of each gene. The alternative activation of each promoter leads to the expression of several isoforms containing a complete N-terminal transactivation domain (TA-isoform) and/or an N-terminally truncated isoform lacking the transactivation domain (Figure 2B). In addition, several COOH-terminus transcripts, originated by alternative splicing, have been identified in p53 family members: α, β, and γ in the case of p53 and p63; α, β, γ, δ, ε, ζ, η, and φ in the case of p73 [15] (Figure 2B).

Since p53, p63, and p73 play a pivotal role in DNA damage response, cell differentiation, proliferation, and death, they currently represent very appealing targets in anticancer drug development [22,23]. Early evidence suggested significant redundancy in the biologic role of the p53 family members. However, additional findings have also indicated non p53-redundant functions for TAp63 and TAp73 as they regulate genes that are not p53 transcriptional targets [24]. Furthermore, while p53 is a tumor suppressor inactivated in half of human cancers, p63 and p73 rarely display mutations in their sequence. It was later demonstrated that TAp63 and TAp73 transactivate distinct but functionally overlapping subsets of known p53-regulated genes involved in cell-cycle arrest and apoptosis [25,26]. It should also be noted that although TAp63 and TAp73 show tumor suppressor properties in different tumors [27,28], several reports suggest an oncogenic function for these proteins as they may inhibit p53 DNA binding activity [29,30,31]. Likewise, the p63 and p73 ΔN variants were considered dominant-negative inhibitors of their respective TA isoforms and of the p53 tumor-suppressor. Indeed, their overexpression in a wide range of tumors is usually associated with an inferior prognosis [17]. However, later experiments also revealed a physiologic role for ΔN isoforms as expression modulators for different p53 family members. For example, p53 and TAp73 are both transcriptional inducers of ΔNp73, thus defining a negative regulatory loop in which p53 and TAp73 activation leads to their functional inhibition by ΔNp73 [26].

In summary, the need to account for a plethora of biological parameters—such as the TA/ΔN p63/p73 ratios, p63/p73 protein-protein interactions, and p63/p73 binding to the promoter of different p53-target genes—has yet to define an unequivocal role for p63 and p73 in human tumorigenesis [16].

### 2.1. p53 and Thyroid Cancer

It is well documented that thyroid carcinoma initiation and progression occurs through the gradual accumulation of multiple genetic alterations. One of the pivotal molecular alterations discriminating ATCs from WDTCs is the inactivation of the *p53* tumor suppressor gene. p53 mutations are common in undifferentiated thyroid tumors (50–80% in ATCs) [32,33]. However, several findings indicate that alterations in the *p53* sequence may also play a role in the early stages of thyroid cancerogenesis. Indeed, p53 mutations were recently found in up to 40% of PTCs and in 22% of oncocytic FTCs [34,35]. Usually, *p53* point mutations are located in the region between exons 5 and 8 [36].

DNA damaging agents activate p53, leading to its binding of specific DNA responsive elements that transcriptionally regulate selected target genes. In thyroid cancer cells, p53 tumor suppressor activity is inhibited by three different mechanisms hindering p53 transcriptional activity, protein stability, and downstream signaling.

**Figure 3:**
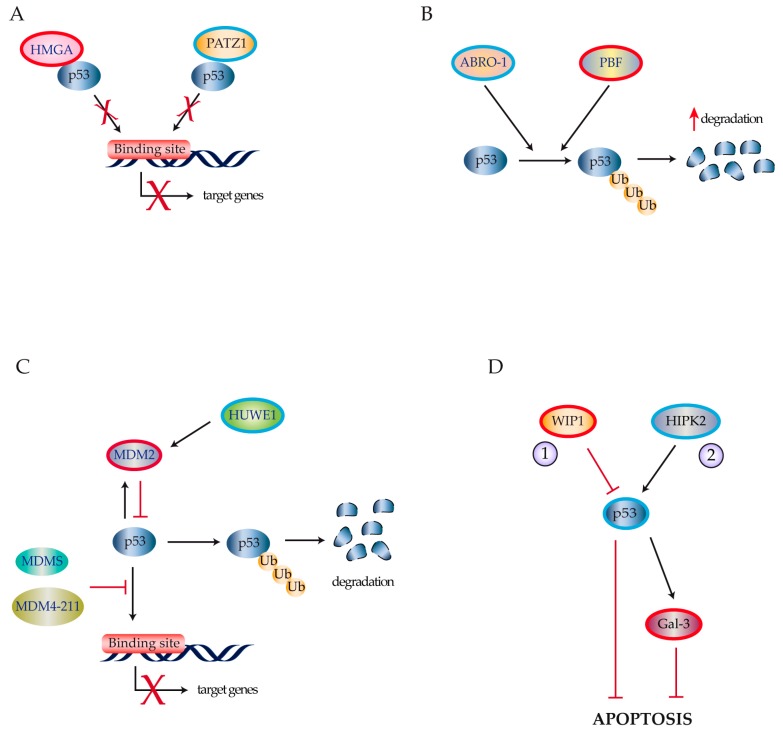
p53 inactivating mechanisms in thyroid cancer. (**A**) HGMA1 over-expression (red) and PATZ1 down-regulation (blue) inhibit p53 ability to bind to its DNA responsive elements; (**B**) ABRO-1 down-regulation (blue) and PBF over-expression (red) decrease p53 stability through an increase in ubiquitin-mediated p53 degradation; (**C**) MDM-S, MDM4-211, and MDM-2 over-expression (red), following ubiquitin E3 ligase HUWE1 down-regulation (blue), inhibit p53 transactivation activity through p53 poly-ubiquitination and by blocking p53 interaction with the DNA binding sites of its target genes; (**D**) Mechanisms leading to reduced apoptotic sensibility in thyroid cancer cells include (1) WIP1 over-expression (red) and (2) decreased HIPK2 expression (blue) causing a reduction of p53 levels (blue) and subsequent increases of Gal-3 (red).

Frasca and colleagues reported that overexpression of high mobility group A factors (HMGA1a, HMGA1b, and HMGA2) functionally disables all p53 family members [15,37] possibly by reducing their DNA-binding activity [37,38,39] (Figure 3A). The POZ/BTB and AT hook containing zinc finger protein (PATZ1) is down-regulated in PDTCs and ATCs. As PATZ1 facilitates p53 binding to its responsive elements. Its down-regulation in thyroid cancer cells reduces p53 biological activity favoring both epithelial-mesenchymal transition and cell migration [40] (Figure 3A).

p53 stability is regulated through a process of ubiquitin-dependent protein degradation. In thyroid cancer cells, the over-expression or down-regulation of different p53 regulatory proteins heavily influence this mechanism.

Zhang et al. reported that Abraxas brother 1 (ABRO1), a component of the BRISC multiprotein complex that specifically cleaves “Lys-63”-linked ubiquitin, is frequently downregulated in thyroid, breast, liver, and kidney tumors [41]. The authors showed that ABRO1 stabilizes p53 by facilitating its interaction with deubiquitinase USP7. Depletion of ABRO1 in thyroid cancer reduces this interaction causing p53 poly-ubiquitination and enhancing cellular transformation of thyroid neoplastic clones [41] (Figure 3B). Likewise, the proto-oncogene PTTG1-Binding Factor (PBF) is highly expressed in WDTC [42], where it interacts with p53 thereby enhancing its poly-ubiquitination, which depends on the E3 ligase activity of MDM2 [43] (Figure 3B).

Murine double minute (MDM) family members are key regulators of p53 expression and function. Both MDM2 and MDM4 negatively regulate p53 [44,45] (Figure 3C). Furthermore, Prodosmo et al. found that MDM-S and MDM4-211—shorter MDM4 spliced variants expressed in thyroid tumors—were strong in vitro p53 inhibitors [46] (Figure 3C). The HECT, UBA, and WWE domain-containing protein 1 (HUWE1) is a ubiquitin E3 ligase for MDM2 that shows deregulated expression in several human cancers. Recently, Ma and colleagues demonstrated that HUWE1 is down-regulated in human thyroid carcinomas thus increasing MDM2 expression and reducing p53 protein stability [47] (Figure 3C).

Alterations in p53 signaling can also contribute to thyroid carcinogenesis by determining checkpoint defects, genomic instability, and inhibition of apoptosis.

Wild type p53 induced phosphatase 1 (WIP1) is a member of the PP2C family of evolutionarily conserved protein phosphatases and is considered a novel proto-oncogene [48,49]. Originally described as a p53-regulated gene, it is overexpressed in several tumors including PTCs where it inhibits p53 as well as p38MAPK and p16 [50] (Figure 3D). Galectin-3 (Gal-3) is an anti-apoptotic molecule that is down-regulated by wild-type p53 [51]. Thyroid tumors usually display low levels of transcription factor Homeodomain Interacting Protein Kinase 2 (HIPK2) that reduces p53 activation resulting in Gal-3 overexpression thus promoting neoplastic transformation [52] (Figure 3D). Zou et al. have recently described an additional mechanism contributing to p53 inactivation in a murine thyroid cancer model. They demonstrated that mice with thyroid-specific expression of the BRAFV600E mutation rapidly developed thyroid carcinomas. In this model, high levels of thyroid-stimulating hormone upregulated the PI3K/AKT pathway thus reducing p53 expression and suppressing BRAFV600E-induced senescence [53]. A further mechanism disregulating p53 downstream signaling involves *FOXE1*, a single-exon coding gene belonging to the forkhead/winged helix-domain protein family. FOXE1 is essential for thyroid gland development and has also been implicated in thyroid cancerogenesis following the discovery of inactivating mutations detected in neoplastic thyrocytes [54,55,56]. Recent evidence suggests that myosin-9 (MYH9) is a binding partner of the papillary thyroid cancer susceptibility candidate 2 (*PTCSC2*) long noncoding RNA. The interaction between MYH9 and PTCSC2 inhibits the *FOXE1* promoter. In turn, lack of FOXE1 results in the transcriptional repression of *THBS1* and *IGFBP3*, pivotal members of the p53 signaling pathway [57].

### 2.2. p63 and Thyroid Cancer

The *p63* gene is infrequently mutated in human cancer. p63 overexpression has been reported in basal and squamous cell carcinomas of the head and neck, in thymomas, basal-like breast cancer, adenocarcinoma of the prostate, and poorly differentiated cervical tumors. p63 may also be aberrantly expressed in thyroid cancer [27,58,59,60].

Several evidences support the tumor suppressor role of TAp63 after interaction with ΔNp63 or other p53 family members. Furthermore, TAp63 induces cell death and suppress metastasis formation by decreasing mobility and invasion [27].

Different studies reported a possible role for p63 in thyrocyte neoplastic transformation [15,30]. Malaguarnera et al. demonstrated that, unlike normal thyroid cells and benign adenomas, most thyroid neoplasias express TAp63α. They also showed that endogenous TAp63α does not play a p53-like role as it fails to induce p21, BAX, and MDM2 and therefore does not cause cell growth arrest and apoptosis. Furthermore, they reported that TAp63α antagonizes p53 by interfering with the binding of more transcriptionally active p63 homologues (TAp63β and TAp63γ) and that of TAp73 [30]. As both TAp63α and ΔNp63α successfully inhibit p53-dependent suppression of colony formation, these findings imply that in thyroid cancer cells both TAp63α and ΔNp63α display a tumor-promoting role [15]. In this respect, Lazzari and colleagues have reported that HIPK2 induces ΔNp63α phosphorylation causing its proteasomal degradation. This mechanism removes the dominant negative effect of ΔNp63α on p53 restoring its pro-apoptotic activity [61].

### 2.3. p73 and Thyroid Cancer

**Figure 4:**
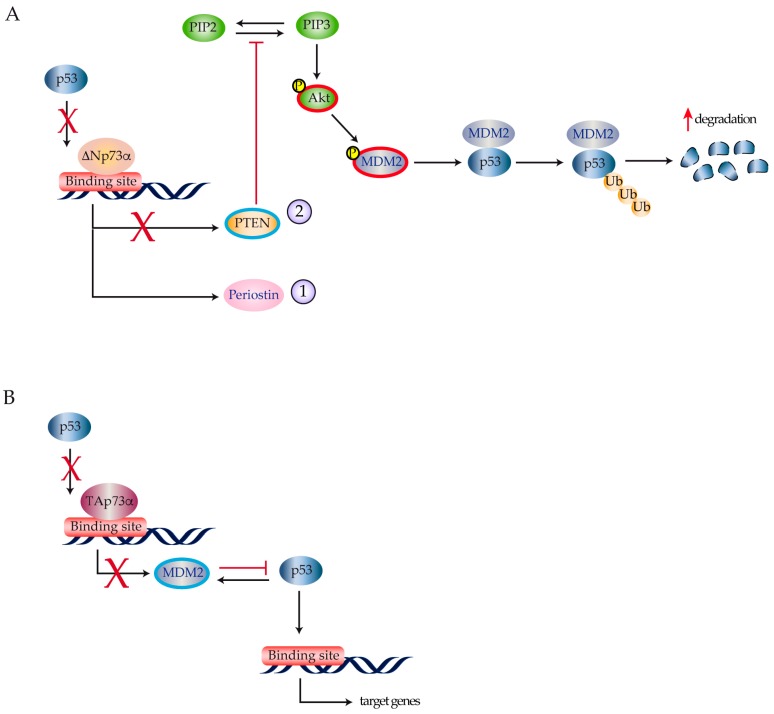
p73 pathway activation in thyroid cancer. (**A**) ΔNp73α binding on the p53 promoter causes the activation of *periostin* (1) and a reduction in *PTEN* expression (blue) (2). Transcriptional repression of the *PTEN* promoter determines an activation of the PI3K-Akt pathway resulting in MDM2 phosphorylation that enhances MDM2-dependent degradation of p53; (**B**) TAp73α blocks p53-dependent transcription on the MDM2 promoter. In turn, this leads to reduced MDM2 expression and increased p53 stability.

Different studies have reported p73 expression in human thyroid tumors, even if the role of p73 in thyroid cancer progression is still controversial [15,62,63]. Both TAp73α and ΔNp73 were detected by RQ-PCR and immunoblots in the large majority of thyroid cancer cell lines isolated from different histotypes (papillary, follicular, and anaplastic), but not in normal cultured thyrocytes [15,64]. Likewise, TAp73 and ΔNp73 transcripts were found in a consistent number of human thyroid carcinomas, although no correlation was found with their clinical and pathological characteristics [65]. Puppin and colleagues published evidence suggesting that ΔNp73α transcriptionally stimulates periostin gene expression in papillary, follicular, and undifferentiated thyroid cancer cells [66] (Figure 4A).

As periostin is associated with accelerated tumor growth, increased neoangiogenesis and higher metastatic potential [67], ΔNp73α expression results in a more aggressive cancer phenotype [68,69]. Vella et al. reported that ΔNp73α represses the *PTEN* promoter [63]. *PTEN* down-regulation increases PIP3 half-life reducing its conversion in PIP2. High levels of PIP3 increase AKT phosphorylation causing MDM2-mediated ubiquitination and p53 degradation [70,71,72] (Figure 4A). *PTEN* repression by ΔNp73α requires binding to a DNA region outside of the canonical p53 site, confirming that p53 and its family members may recognize different sequences on the same promoter. In this scenario, the functional interactions among p53 family members may be either synergistic or antagonistic with respect to tumor suppression. This is in line with previous reports showing that, in different thyroid cancer models, co-expression of p53 and TAp73α may alternatively result in TAp73α down-regulation [62] or stronger p53 tumor suppressor activity [73]. In the latter case, TAp73α inhibits MDM2-mediated p53 protein degradation by reducing MDM2 binding to p53 and by directly antagonizing p53 activity on the *MDM2* promoter (Figure 4B).

## 3. Novel Therapeutic Approaches for Undifferentiated Thyroid Cancer

The aforementioned advances in understanding the contribution of p53 family members to the pathogenesis of thyroid cancer have provided several opportunities for the use of molecular targeted therapies.

**Figure 5:**
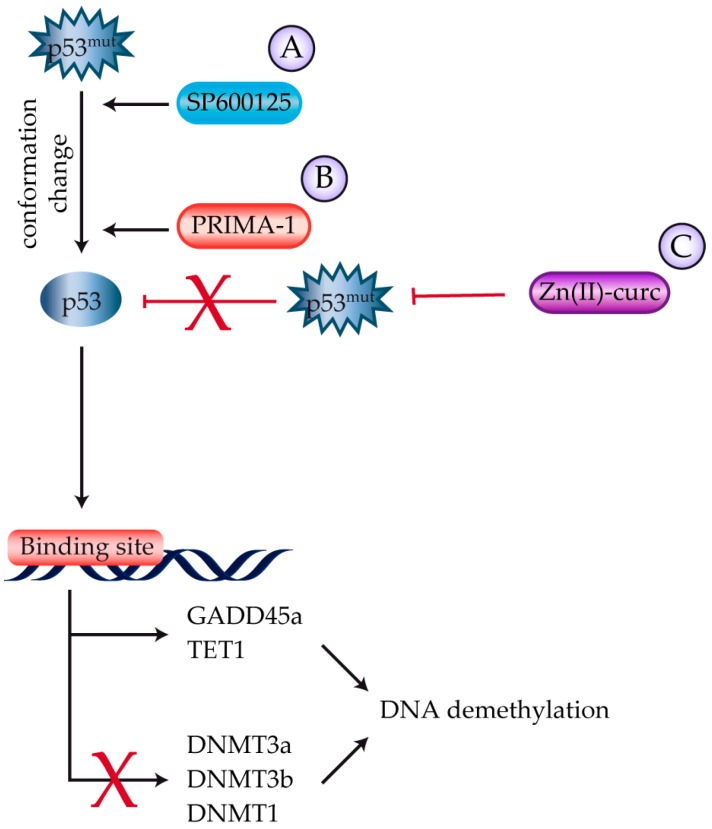
Strategies to reactivate p53 in thyroid cancer. (**A**) SP600125 and (**B**) PRIMA-1 reactivate mutant p53 via conformational changes. In thyroid cancer, modifications caused by PRIMA-1 induce global DNA demethylation through the up-regulation of GADD45a and TET1 and down-regulation of DNMT1, 3a, and 3b; (**C**) Zn(II)-curc reduces mutant p53 expression by restoring wild-type p53-DNA binding activity to target gene promoters.

Multiple clinical trials using various multi-kinase inhibitors (MKIs) have led to the FDA and EMA approval of Sorafenib [74] and Lenvatinib [75] for undifferentiated thyroid cancer. Grassi and colleagues have also investigated SP600125, a reversible ATP-competitive MKI with anticancer properties against undifferentiated thyroid cancer. The compound reduces cell migration while killing human thyrocytes through the activation of mutant p53 and the concomitant inhibition of the ROCK/HDAC6 pathway [76] (Figure 5).

Besides MKIs, additional strategies for thyroid cancer treatment are currently being investigated that employ strategies that modulate epigenetic changes in thyroid cancer DNA, restore the transcriptional activity of mutant p53, and block signal transduction downstream of different p53 family members.

Studies on histone post-translational modifications that support tumor development and progression have focused on DNA methyltransferases (DNMT) inhibitors such as 5′-azadeoxycytidine with the aim of enabling thyroid cancer differentiation, thus restoring its sensitivity to radioactive iodine treatment [77]. This effect has also been investigated with the MEK1/MEK2 inhibitor Selumetinib [78]. Thailandepsin A (TDP-A) is a novel class I histone deacetylase inhibitor that induces a dose- and time-dependent anti-proliferative effect on human thyroid cancer cells mainly attributed to activation of the extrinsic apoptotic pathway coupled with cell cycle arrest [79]. Proteasome inhibitors such as Bortezomib have also been tested on thyroid cancer cells leading to significant inhibition of neoplastic thyrocyte proliferation [80,81].

p53 reactivation and induction of massive apoptosis (PRIMA-1) is a compound that reactivates the DNA binding ability of mutant p53 by restoring its conformation through two different mechanisms: (i) it induces Hsp90 over-expression thus enhancing its binding to mutant p53 and (ii) it covalently binds to mutant p53 [82]. Qiang and colleagues have recently defined the mechanism responsible for PRIMA-1-dependent rescue of mutant p53 function in thyroid cancer cells [83]. They showed that PRIMA-1 causes global DNA demethylation in cancer cells expressing mutant p53 mainly through inhibition of DNMT 1, 3a, and 3b, and upregulation of GADD45a. PRIMA-1 also increased the expression of the ten-eleven translocation (TET) family of 5mC-hydroxylases, particularly TET1, further contributing to DNA demethylation. Messina et al. studied the effect of PRIMA-1 in thyroid cancer cell lines with both wild-type and D259Y and K286E p53 mutants. They found that PRIMA-1 successfully killed undifferentiated thyroid carcinoma cells carrying mutant p53 that were refractory to chemotherapy [84] (Figure 5).

A curcumin-based zinc compound [Zn(II)-curc] also reduced mutant p53 expression in thyroid cancer cells, reactivating p53 tumor suppressor activity and resulting in increased response to anti-cancer therapies [85] (Figure 5).

Finally, the mammalian target of rapamycin (mTOR) pathway inhibitor Everolimus has been studied in a phase II trial of 40 patients with locally advanced or metastatic thyroid cancer [86] producing stable disease in 29 (76%) patients and a median Progression Free Survival of 47 weeks (95% CI 14.9–78.5).

## 4. Conclusions

The concept of precision medicine postulates that effective treatment strategies should be tailored to the individual variability of both the patient and her/his disease. While alterations in p53 sequence, stability, and downstream signaling are heavily involved in thyroid carcinogenesis, the role of the remaining p53 family members in the development and progression of thyroid cancer has yet to be fully elucidated. Nevertheless, increasing evidence indicates that p53 family members contribute to the development of multiple thyroid cancer variants and an ever-increasing number of therapeutic molecules targeting these proteins may soon be available in the clinical setting.

## Abbreviations

WDTC	Well Differentiated Thyroid Cancer
PDTC	Poorly Differentiated Thyroid Cancer
ATC	Anaplastic Thyroid Cancer
RET	Rearranged During Transfection
PTC	Papillary Thyroid Cancer
FTC	Follicular Thyroid cancer
MAPK	Mitogen-Activated Protein Kinase
PPARc	Peroxisome Proliferator-Activated Receptor C
PAX8	Paired Box 8
RAS	Rat Sarcoma
PI3K	Phosphatidyl-Inositol-3-Kinase
PTEN	Phosphatase and Tensin Homolog
CTNNB1	Catenin beta 1
TAD	Transactivation Domain
DBD	DNA Binding Domain
OD	Oligomerization Domain
PRD	Prolin-Rich Sequence recognition Domain
SAMD	Steril Alpha Motif Domain
TID	Transactivation Inhibitory Domain
HMGA	High Mobility Group A
PATZ1	POZ7BTB and AT Hook containing Zinc-finger Protein
ABRO1	Abraxas Brother 1
PBF	PTTG1-Binding Factor
MDM	Murine Double Minute
HUWE1	HECT, UBA, and WWE Domain-Containing Protein 1
WIP1	Wild Type p53 Induced Phosphatases 1
Gal-3	Galectin-3
HIPK2	Homeodomain Interacting Protein Kinase 2
FOXE1	Forkhead7Winged Helix-Domain Protein
MYH9	Myosin-9
PTCSC2	Papillary Thyroid Cancer Susceptibility Candidate 2
THBS1	Thrombospondin 1
IGFBP3	Insulin-Like Growth Factor Binding Protein 3
MKIs	Multi-Kinase Inhibitors
HDAC	Histone Deacetylase
ROCK	Rho Kinase
DNMT	DNA Methyltransferase
MEK	Mitogen-Activated protein kinase kinase
TDP-A	Thailandepsin A
PRIMA-1	p53 Reactivation and Induction of Massive Apoptosis
GADD45A	Growth Arrest and DNA Damage Inducible Alpha
TET	Ten-Eleven Translocation
mTOR	Mammalian Target of Rapamycine
